# Generation of subject-specific, dynamic, multisegment ankle and foot models to improve orthotic design: a feasibility study

**DOI:** 10.1186/1471-2474-12-256

**Published:** 2011-11-10

**Authors:** Michiel Oosterwaal, Scott Telfer, Søren Tørholm, Sylvain Carbes, Lodewijk W van Rhijn, Ross Macduff, Kenneth Meijer, Jim Woodburn

**Affiliations:** 1 NUTRIM, Department of Human Movement Sciences, Maastricht University Medical Centre +, PO 5800, 6202 AZ Maastricht, The Netherlands; 2 CAPHRI, Dep. of Orthopaedic Surgery, Maastricht University Medical Centre +, PO 5800, 6202 AZ Maastricht, The Netherlands; 3 School of Health and Life Sciences, Glasgow Caledonian University, Cowcaddens Road, Glasgow, UK; 4 AnyBody Technology A/S, Niels Jernes Vej 10, DK-9220 Aalborg East, Denmark; 5 Department of Radiology, Glasgow Royal Infirmary, Glasgow, UK

## Abstract

**Background:**

Currently, custom foot and ankle orthosis prescription and design tend to be based on traditional techniques, which can result in devices which vary greatly between clinicians and repeat prescription. The use of computational models of the foot may give further insight in the biomechanical effects of these devices and allow a more standardised approach to be taken to their design, however due to the complexity of the foot the models must be highly detailed and dynamic.

**Methods/Design:**

Functional and anatomical datasets will be collected in a multicentre study from 10 healthy participants and 15 patients requiring orthotic devices. The patient group will include individuals with metarsalgia, flexible flat foot and drop foot.

Each participant will undergo a clinical foot function assessment, 3D surface scans of the foot under different loading conditions, and detailed gait analysis including kinematic, kinetic, muscle activity and plantar pressure measurements in both barefoot and shod conditions. Following this each participant will undergo computed tomography (CT) imaging of their foot and ankle under a range of loads and positions while plantar pressures are recorded. A further subgroup of participants will undergo magnetic resonance imaging (MRI) of the foot and ankle.

Imaging data will be segmented to derive the geometry of the bones and the orientation of the joint axes. Insertion points of muscles and ligaments will be determined from the MRI and CT-scans and soft tissue material properties computed from the loaded CT data in combination with the plantar pressure measurements. Gait analysis data will be used to drive the models and in combination with the 3D surface scans for scaling purposes. Predicted plantar pressures and muscle activation patterns predicted from the models will be compared to determine the validity of the models.

**Discussion:**

This protocol will lead to the generation of unique datasets which will be used to develop linked inverse dynamic and forward dynamic biomechanical foot models. These models may be beneficial in predicting the effect of and thus improving the efficacy of orthotic devices for the foot and ankle.

## Background

It has been estimated that almost 200 million people in Europe have disabling foot or ankle pain and that this figure will rise with aging societies and the associated increase in prevalence of chronic long term conditions [1-5]. Foot pain can cause loss of function, discomfort, and a general lowering of the patient's quality of life. Custom ankle-foot and foot orthoses are a popularly prescribed conservative treatment intended to alleviate this pain, via a number of purported mechanisms. Currently the design of these devices is largely based around capturing the foot shape using traditional techniques such as plaster casting, and determining abnormal foot function through clinical examination. These approaches may lead to variability in the prescription and restrict design choice and personalised function to simple parameters such as cushioning, support and range of motion control.

Computational modelling of the human body - ranging from the force interactions of joints to the way cells communicate with each other - has advanced significantly in the past few decades and it is now an important and useful tool for researchers and clinicians. These models provide a method of simulating and assessing interventions that are being developed, reducing the time and risk involved with trialling in humans. This approach is particularly appealing for studying foot biomechanics due to the challenging nature of directly investigating the internal loading and movements of the complex structure of bones and soft tissues of the foot that occur during gait.

A small but growing body of research, primarily based around finite element (FE) analysis, has studied the foot using models based on different combinations of gait analysis, pressure distribution measurements, computed tomography (CT) and magnetic resonance (MR) imaging of the foot. This has provided insights into inflammation of the plantar fascia [6,7], pressure assessment of the diabetic foot [8] and therapeutic footwear [9].

Lower limb musculoskeletal biomechanics is an area where extensive modelling work has been successfully carried out, supporting the development and assessment of a range of treatments and interventions [10-13]. In terms of the foot however, these models have tended to represent it as a single rigid segment, and it is only recently that progress has been made in incorporating some of the intrinsic joints of the foot [14].

With these factors in mind, it is suggested that there is potential for highly detailed biomechanical foot models to be used in the process of designing orthotic interventions for the foot and ankle. These could lead to the development of devices and prescription paradigms which could improve the efficacy of these devices and benefit the patient, as well as reducing long term treatment costs for the healthcare provider.

### Models

This article describes a protocol that has been developed to generate the biomechanical and anatomical data required to produce two linked foot models: a forward dynamic model that combines a multibody approach with FE analysis; and an inverse dynamic model which describes the musculoskeletal interactions of the system. The forward dynamic model will be developed using the Madymo software platform (TASS, Rijswijk), and the inverse dynamic model in the AnyBody modelling system (AnyBody Technology, Aalborg). Generally, forward dynamic models need joint torques and/or muscle forces as an input to compute kinematics, and for a complex structure like the foot this information can be generated from inverse dynamic models driven by motion capture data. Using the same dataset to construct both models leads to the possibility of a combination of both models in a final application.

#### Inverse dynamic model

**Figure 1:**
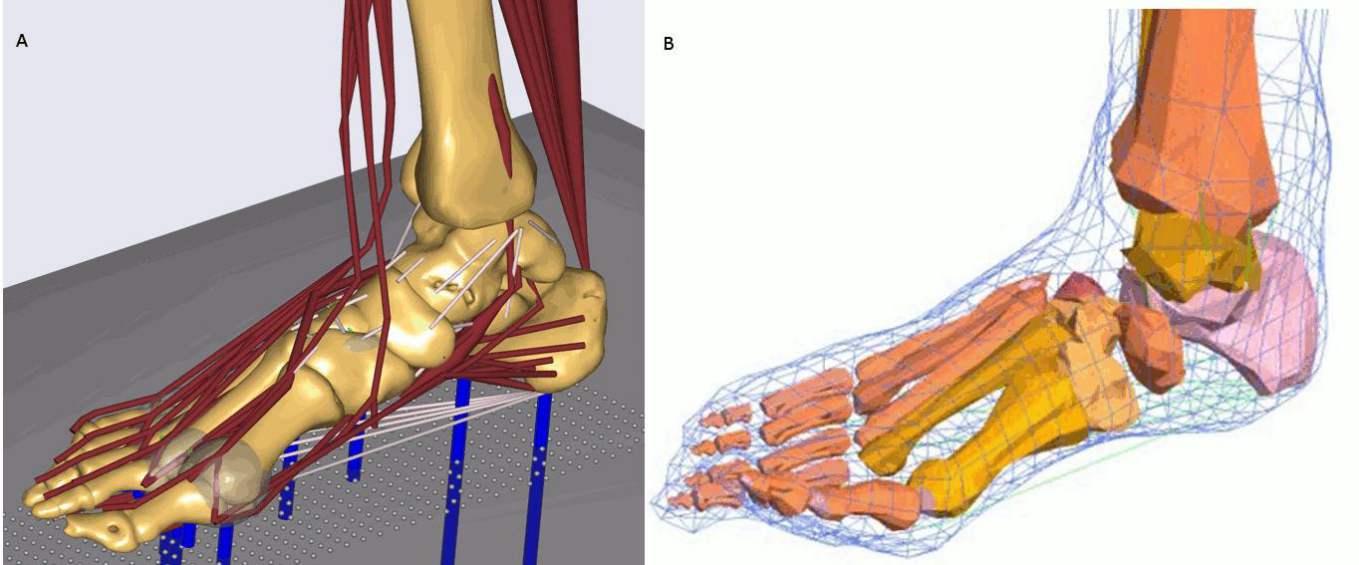
**Foot models**. Graphical representation of a) Current inverse dynamic model, b) Current forward dynamic model

Standard gait analysis and inverse dynamic models of the lower extremity consider the foot as a single rigid segment. Some models have been developed that describe the foot in more detail, however to the authors' knowledge no kinematic model has attempted to describe all 26 bones. The proposed kinematic model is scalable and parametric and will integrate into the existing AnyBody whole body musculoskeletal model (Figure 1). The model will contain all of the ligaments and muscles of the foot and ankle. By combing the inverse dynamic modelling with an optimisation algorithm the model will provide insight in function of the foot and leg muscles during gait.

#### Forward dynamic model

The forward dynamic model is a combined multibody and FE approach. Bones, joints, ligaments and muscles are defined as multibody elements. This multibody model is surrounded by a freeform FE sheet, representing the skin. The soft tissue characteristics are implemented by loading functions that connect the sheet to the multibody elements. This combination leads to a computationally less complex model compared to a full FE model of the foot and ankle. This reduction could be improved by a full multibody representation of the ankle-foot complex, however in this model computation of the plantar pressure would be impossible. In the proposed model this is solved by a FE mesh representing the plantar surface, leading to the possibility of performing a dynamic simulation to compute the deforming plantar surface.

An existing model (Figure 1) has previously been developed from a combination of data from a post-mortem human subject and information derived from existing literature [15]. The generation of one complete dataset leads to a consistent model. Beyond this, the generation of datasets from several subjects creates the possibility of scaling the model. This scaling will be extended for patients requiring foot and ankle orthoses.

The overall aim of this work is to develop detailed and accurate biomechanical models of the foot and ankle which can be used to inform the design of foot and ankle orthoses by predicting the biomechanical effects of the device.

## Methods/Design

### Study Design

This study is a feasibility/pilot study to generate data that will be used to develop the biomechanical foot models described in the previous section. It is a multicentre study with data being collected at the motion capture lab of Glasgow Caledonian University (GCU) and Radiology Department of Glasgow Royal Infirmary, and at the motion capture lab of Maastricht University Medical Centre (MUMC+) and Department of Radiology at MUMC+. The data collection will take a period of six months for each centre. Data collection will last approximately four hours for each subject. The data acquisition will be independent, and after acquisition the data will be pooled and used to develop both models.

### Ethical Consideration

This study will be conducted in accordance to the Declaration of Helsinki. Ethical approval for this study has been granted by the West of Scotland Research Ethics Committee (application reference 10/S1001/24) and National Health Service Greater Glasgow and Clyde Research and Development Committee (reference GN10RH187) for the UK site. At the Dutch centre, the study was granted approval by the Medical Ethical Committee azM/UM (reference number NL31656.068.10/MEC 08-2-028).

### Participants

Two groups of participants will be investigated, healthy individuals and patients with foot and/or ankle problems. Healthy volunteers will be recruited via convenience sampling from staff bodies of the test centres. Participants with pathological foot problems will be informed about the study by their orthopaedic surgeon (MUMC+) or podiatrist (GCU) during the course of attending a routine appointment at a local foot and ankle clinic. Table 1 gives an overview of the study population.

**Table 1:** Study population

	Total	Maastricht	Glasgow
Healthy subjects	10	5	5
Patients requiring:	15	7	8
*Pressure releasing orthotics*	7	3	4
*Alignment improving orthotics*	7	4	3
*Ankle foot orthosis for motion control*	1	0	1

#### Inclusion criteria

In both groups, healthy feet and group pathological feet, participants will be included if they are physically able to walk at least 20 meters barefoot and unaided. In addition, participants must be in age group 18-50 years and have feet of size 38-44 (EUR). All participants will be fully competent and be able to give informed consent.

In addition to this general inclusion criteria, participants in the patient group must fall into one of the following categories: 1) Patients with metatarsalgia on their right foot, who would be prescribed pressure relieving foot orthoses. Metatarsalgia will be clinically diagnosed as the presence of one or more features of spontaneous pain or tenderness at one or more metatarsophalangeal joints elicited by firm pressure and/or movement of the joint. 2) Patients with flexible flat foot deformities on their right foot, who would be prescribed foot orthoses to improve alignment. For the purposes of this study, flexible flat foot is diagnosed as a correctable relaxed calcaneal stance position greater than six degrees everted with a navicular tuberosity height lower than 35 mm. 3) Hemiplegic stroke patients who would be prescribed an ankle foot orthosis on their right foot to control the motion of their ankle during gait.

#### Exclusion criteria

Participants in both groups will be excluded if they have a diagnosable disease with known involvement of the lower limb and foot including, for example, diabetes mellitus, peripheral vascular disease and rheumatoid arthritis. Pregnant and lactating women will not be eligable for participation due to the radiation dose associated with the CT scans that are part of this protocol.

To be eligible for inclusion in the healthy group, participants must not currently be receiving treatment for any foot or ankle conditions or have any significant history of foot or ankle trauma, injury, fracture or dislocation.

### Clarification of sample size

The exploratory nature of this study makes it difficult to calculate power requirements for statistical purposes. Therefore, a pragmatic approach has been taken with the number of subjects chosen by the opinion of experts to cover a broad scope of variation of foot problems. A broad range of subjects in terms of foot size, age, BMI, will be included for development of scaling methods and to test the validity of the models.

### Study procedure

Participants will initially be asked to attend to the motion capture laboratory at GCU or MUMC+. Each participant will undergo a clinical foot assessment, 3D surface scanning of the foot and gait analysis. Each participant will then undergo a set of CT scans and a subset of participants, the five healthy subjects tested at GCU, will also undergo MRI scans of the foot at a nearby medical imaging facility.

The full protocol will last approximately four hours. Research teams at both institutions have extensive experience of collecting and processing the functional and imaging measurements required for this study. At GCU, the clinical assessment will be carried out by a clinician with over 20 years' experience. At MUMC+, the clinical assessment will be carried out by a clinician with over 5 years' experience.

### Clinical foot assessment

Each participant will first have an extended clinical foot function assessment during which range of joint motion, muscle strength, posture and impairments such as pain, stiffness and deformity will be recorded. The clinician will also assess the participant's ability to carry out the tasks required during the remainder of the protocol, particularly in the case of those with neuromuscular conditions. Each participant will complete the Manchester Foot Pain and Disability Questionnaire [16] and the foot-function index [17,18].

### 3-D surface geometry

Each participant will be asked to stand with their right foot in a 3D foot surface scanner (Easy Foot Scan; OrthoBaltic, Kaunas, Lithuania) and scans will be taken with: minimal weight on the foot (< 5% body weight); 50% body weight on the foot; and > 95% body weight on the foot. The participant will be asked before each scan if they are comfortable maintaining the related level of weight bearing on the foot and the scan will only be carried out if they are able to maintain this load. These scans will take approximately one minute each including positioning. Supports will be provided and a researcher will be nearby to reduce the risk of falls.

### Gait analysis

Each participant will undergo a comprehensive assessment of their gait, in both barefoot and shod conditions. During gait analysis, kinematic, kinetic, electromyographic (EMG) and plantar pressure measurements from the participant's right foot and leg will be collected simultaneously during the stance phase of gait.

#### Kinematic measurements

Kinematic data will be collected using a 12 camera Qualisys system (Glasgow) and an eight camera Vicon system (Maastricht). Residual errors of < 1 mm are deemed acceptable for both systems.

Bony and tracking landmarks (see Table 2) will be identified through physical palpation of the relevant areas on the foot and leg by trained researchers. Once identified, these points are indicated on the skin with non-permanent marker. Passive, reflective markers (Qualisys AB, Gothenburg, Sweden) will be attached at these points using double sided tape. The marker model used is an adapted version of that used in the multi-segment foot model described in Hyslop et al (2010) [19]. The model has been extended with additional markers on the thigh, hips, lesser toes and the lateral cuneiform.

**Table 2:** Kinematics

Number	Landmark/Location	Label Name	Marker Size (mm)	Barefoot trials only (B) or shod and barefoot (S)
1	Right iliac crest	RAIC	19	S
2	Left iliac crest	LAIC	19	S
3	Right posterior superior iliac spine	RPSI	19	S
4	Left posterior superior iliac spine	LPSI	19	S
5	Right greater trochanter	RGT	12	S
6	Left greater trochanter	LGT	12	S
7	Thigh 1^st^	THI1	19	S
8	Thigh 2^nd^	THI2	19	S
9	Lateral knee	LKNE	12	S
10	Tibial tuberosity	TTUB	7	S
11	Head of fibula	HFIB	7	S
12	Shin 1^st^	SHN1	19	S
13	Shin 2^nd^	SHN2	19	S
14	Superior calcaneum	SCAL	7	S
15	Inferior calcaneum	ICAL	7	S
16	Medial malleolus	MMAL	7	S
17	Medial calcaneum	MCAL	7	S
18	Tuberosity navicular	NAV	7	S
19	Proximal 1^st ^met head	P1MT	7	B
20	Central 1^st ^met	C1MT	7 (on 20 mm wand)	B
21	Medial 1^st ^met head	M1MT	7	S
22	Lateral 1^st ^met head	L1MH	7	B
23	Hallux 1^st^	HLX1	7	B
24	Hallux 2^nd^	HLX2	7	B
25	Hallux 3^rd^	HLX3	7	B
26	Lateral malleolus	LMAL	7	S
27	Lateral calcaneum	LCAL	7	S
28	Cuboid	CUB	7	B
29	Proximal 5^th ^met	P5MT	7	S
30	Distal 5^th ^met	D5MT	4	S
31	Intermediate cuneiform	ICUN	7	B
32	Lateral cuneiform	LCUN	7	B
33	2^nd ^met head	D2MT	4	B
34	3^rd ^met head	D3MT	4	B
35	4^th ^met head	D4MT	4	B
36	2^nd ^proximal phalanx	D2PP	4	B
37	3^rd ^proximal phalanx	D3PP	4	B
38	4^th ^proximal phalanx	D4PP	4	B
39	5^th ^proximal phalanx	D5PP	4	B
40	2^nd ^distal phalanx (on nail)	D2DP	4	B
41	3^rd ^distal phalanx (on nail)	D3DP	4	B
42	4^th ^distal phalanx (on nail)	D4DP	4	B
43	5^th ^distal phalanx (on nail)	D5DP	4	B

Overview of the kinematic marker set

For the shod trials, the participants are provided with standardised footwear (Flextop Diabetic Black shoes, Reed Medical, Blackburn, UK). A number of the foot mounted markers used in the barefoot trials will be removed for the shod trials (see Table 2 for details), and holes are cut into the shoes to allow the remaining markers to be visualised by the motion capture system and to move during walking without interference.

#### Kinetic measurements

Kinetic measurements will be taken at both centres using Kistler force plates (Kistler Instrument Corp., Amherst, NY) synchronised with and recorded through the QTM software (GCU) or Nexus software (MUMC+) at a frequency of 2400 Hz.

#### Electromyographic measurements

All parts of the protocol relating to surface EMG measurement will be carried out in accordance with the guidelines produced by the Surface Electromyography for the Non-Invasive Assessment of Muscles (SENIAM) project [20]. These guidelines cover the location and orientation of electrode placement, skin preparation and signal tests for each muscle.

Trigno wireless EMG systems (Delsys Inc, Boston, MA) will be used to collect the EMG measurements at both centres. The electrode units will be attached to the following muscles: tibialis anterior, gastrocnemius medialis, gastrocnemius lateralis, soleus, peroneus longus, vastus lateralis, rectus femoris, and biceps femoris. Signals from each muscle will be checked in real time using EMGworks software (Delsys Inc, Boston, MA) while performing the exercises described in the SENIAM guidelines.

Reference measurements will be taken for each muscle in the form of maximal voluntary isometric contractions (MVICs). Measurements will be recorded for five seconds in total with the participant being asked to gradually build up the force they apply over the first two seconds, and maintain their maximum effort for the remainder of the contraction. Each contraction is repeated three times in a non-consecutive randomised order with at least one minute recovery time between exercises.

For the MVIC and gait components of the testing, EMG signals from the Trigno sensors will be recorded through the analogue channels of QTM or VICON software at a frequency of 2400 Hz.

#### Plantar pressure measurements

For barefoot trials, plantar pressure measurements will be collected using a 0.5 m Footscan^® ^plate (RSscan International, Lammerdries, Belgium) recording at 500 Hz. The plate is mounted directly on top of the force plate and secured in place using double sided tape. The effect of this setup on the accuracy of the force plate was assessed using the CalTester^® ^quality assurance tool (C-Motion Inc, Germantown, MA) and errors were found to be within acceptable limits. To ensure high levels of accuracy in the pressure measurements, the pressure plate is dynamically calibrated with the vertical force signals from the force plate.

In-shoe plantar pressure measurements will be made using the Pedar^® ^system (Novel, GmbH, Munich, Germany) at 50 Hz. In addition, pressure under the sole of the shoe will be recorded during these trials using the Footscan^® ^plate.

#### Testing

A static trial will be recorded with the participant in a relaxed standing pose in the motion capture area. The participant will then be asked to walk barefoot at a self selected speed along the motion capture area such that their right foot strikes the centre of the pressure plate. Five successful walking trials will be recorded. This will then be repeated for the shod trials.

### Imaging

#### Computed tomography (CT)

CT scans of the leg and foot will be undertaken in hospital radiology centres. Each participant will undergo four scans under a variety of different conditions. First the knee, shank and foot will be scanned with the participant asked to apply only minimal loading to the plate with the foot in a neutral position (90 degrees flexion). Then three scans imaging the foot and ankle only will be taken in a randomised order: foot loaded to 50% body weight with the foot in the neutral position; foot loaded to 50% body weight with the foot 25° of plantar flexion; 50% body weight with 10° of dorsiflexion.

**Figure 2:**
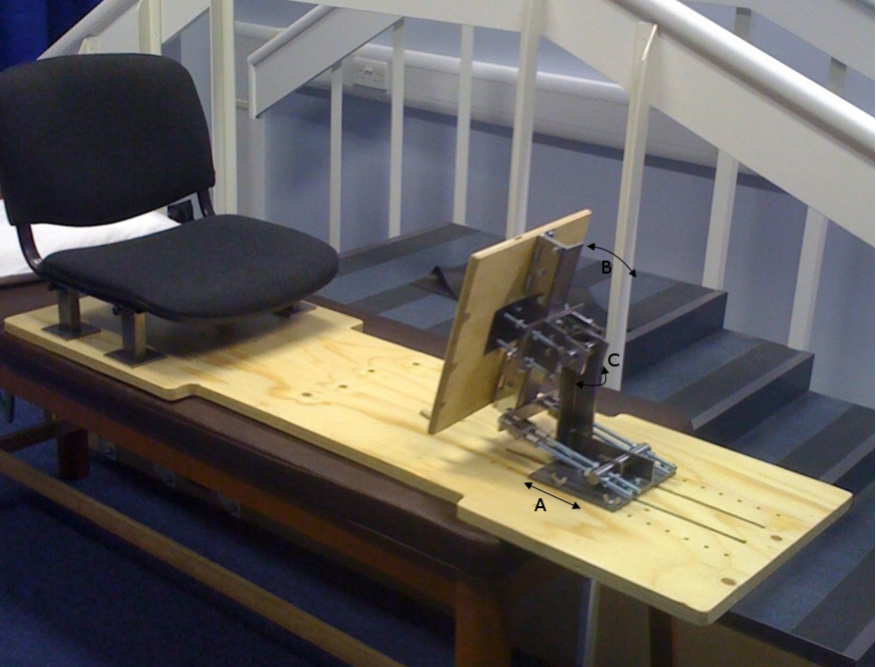
**Loading rig**. A: loading plate can be moved linearly to accommodate different leg lengths; B: plate can be tilted to give plantar/dorsi flexion of the foot; C: plate can be tilted to invert/evert the foot.

To allow the participant to apply force on the foot while being scanned, a novel loading rig was developed and fabricated (Figure 2). Previously, several investigators have described similar systems (14) but to the authors' knowledge this is the first device that allows the position of the foot in relation to the lower leg to be easily and quickly manipulated.

The loading rig takes the form of a chair fixed on to a plywood base plate and a plate for the participant to push against with their foot at the other end. The nature of CT means that metallic objects can cause interference in the image, an effect known as scattering. To avoid this, the steel components which allow the loading plate to be repositioned are kept behind the scan plane. The loading plate can be easily moved closer to or further from the chair at fixed 20 mm intervals to suit the participant's leg length. A standard bathroom scale with a large LED display is mounted on the loading plate and is used to provide feedback to the participant on the level of loading being applied. The relatively short time frame of the CT scan (5-10 seconds) allows medium level loads to be applied and maintained over its duration. During the scans, a Pedar pressure insole (Novel, GmbH, Munich, Germany) is placed between the foot and the loading plate. In addition, using the pen marks made on the foot to guide placement, radiopaque markers (4 mm diameter Beekley Spots^®^, Oncology Imaging Systems, East Hoathly, UK) are attached at the same points as the motion capture markers during gait analysis.

The following parameters were used to acquire the four scans on an Aquilion 64 slice scanner (Toshiba, Tokyo, Japan) at the Glasgow centre or a Brilliance 64 slices (Phillips, Amsterdam, Netherlands) at Maastricht: 120 kVp, 100 mAs, 1.0 mm collimination, 1.0 mm effective slice thickness, pitch factor 41, rotation time 0.5 seconds, B30S medium smooth reconstruction kernel, 512 × 512 matrix.

#### MR

Due to the limitations of soft tissue information that can be inferred from CT imaging, a subset of five participants will also have MR scans taken of their right foot. This will take place on a different day to the rest of the testing, but within six weeks of the initial assessment.

For the MR scans the foot is placed in a suitable imaging coil and foam padding is placed around the foot to prevent movement during the scan. Images will be acquired using a 3T system (Siemens Verio; Erlangen, Germany). Three scans will be taken in total, a T2-weighted scan covering the full foot and ankle, and two T1-weighted scans, one of the rearfoot/midfoot complex and one of the forefoot/midfoot complex.

Scanning parameters for the T2 scan (trueFISP 3D volume) are: repetition time, 9.8 ms; echo time, 4.92 ms; flip angle, 35°; field of view, 290 mm; slice thickness, 0.6 mm (no slice gap); slices per slab, 144; matrix, 256 × 216 (interpolated); phase encoding, anterior to posterior; number of averages, 2.

Scanning parameters for the T1 scan (Space 3D volume) are: repetition time 700 ms; echo time 22 ms; flip angle 105°; field of view, 150 mm; slice thickness 0.8 mm (no slice gap); slices per slab, 94; matrix 320 × 290 (interpolated); phase encoding anterior to posterior; averages, 2.4.

### Data Processing

#### Gait analysis data

The kinematic data, the kinetic data and the plantar pressure will be processed by using Nexus (Vicon, Oxford, UK) software (Maastricht) or Qualisys Track Manager (Qualisys, Gothenburg, Sweden) software (Glasgow) and saved in the C3D format.

Kinematic and kinetic measurements will be used as an input for the inverse dynamic model and to validate the forward dynamic model. The dataset of one healthy subject will be used to develop the first model. Afterwards the datasets of the other healthy subjects will be used to refine the initial model, develop kinematic and kinetic rules and a morphing-based scaling facility so the models can be personalised. After the development of the morphing algorithm the combination of this algorithm and the two models will be used to predict the effect of insoles.

Data from the foot assessments in combination with the 3D surface scans will be processed to develop an algorithm for personalisation of the models with a non invasive, low-end method. It is intended to investigate if an algorithm can be developed to drive the musculoskeletal model directly from plantar pressure measurements, allowing the kinematic parameters of the lower extremities to be determined without the need for a full motion capture system.

#### Imaging data

CT data will be segmented into the individual bones of the foot using Mimics (Materialise, Leuven, Belgium) image processing software. The CT-data will be used to compute the joint axes of the foot, by correlating the positions of the bones in the various positions. The loaded CT-data in combination with the pressure measurement will be used to gain insight in the soft tissue characteristics of the foot. Insertion and via points will be identified from partial segmentation of the MR data and described as co-ordinates on the bones segmented from the CT data. Insertion points will be defined as the centre of the area of insertion.

### Data Analysis

Initial validation of the inverse dynamic model will be carried out by visual inspection of predicted and measured (via EMG) muscle timings. Formal validation of both models will be carried out by comparing plantar pressure measurements taken during gait analysis to those predicted by the forward dynamic model. Intraclass correlation coefficients (ICC_2, k_) will be used to compare peak and average pressures at the hallux, the lesser toes, each of the metatarsal heads, the midfoot region, and the lateral and medial heel. Future studies will investigate the validity of the models for predicting biomechanical changes induced by orthoses.

## Discussion

The primary aim of this study is to collect data for the development of two biomechanical foot models. A single dataset that includes a clinical foot assessment, gait analysis and imaging data has, to the authors' knowledge, not been combined previously for the generation of a biomechanical foot model [21]. The geometry of existing FE models tend to be based on an MRI [22] or CT dataset [23] for one subject, while material properties are obtained from the literature and describe averages of larger groups of subjects. Cheung et al [24] have used an FE model to simulate several phases of the gait cycle, deriving boundary conditions for these static simulations from EMG and ground reaction force measurements.

To the authors' knowledge, multibody models of the foot describing the level of detail proposed here have not previously been attempted. An inverse dynamic model with three segments has been developed by Saraswat et al. [14], however the anatomical information was derived from literature, making it impossible to produce a personalised model.

Various FE models have been used in combination with insole models [25-32]. These models have been developed in varying complexity, from 2D FE simulations of the second ray [26] to the inclusion of nonlinear material properties [27]. These studies are mainly parametric studies in which several properties of the insole are simulated in a static simulation of the mid-stance phase. Variations can be made in geometry [27] or material properties [25], or a combination [28]. The computational complexity of highly detailed FE models however, means that only a static simulation can be run.

A driving input of the models that will be developed in this study is the kinematic data. This data is acquired by a set of skin mounted markers. Markers are positioned on bony-landmarks, taking into account the influence of muscles, tendons and ligaments on skin motion. Previously reported bone pin studies show the distinction between the movements of the skin markers and the bones [33]. This distinction has two causes: soft tissue movement [34] and the rigid body violation [35]. The latter point is solved by introducing a 26 segment model. The problem of soft tissue movement is partially addressed by performing loaded CT with radio-opaque markers and having the option to include direction dependent weighting factors in the AnyBody model. A bone pin study involving all bones would be difficult due to the small size of the bones. In addition, the confounding effect of this invasive method on the motion pattern of gait is not known.

Validation of the models is partly performed by comparison of the muscle activity patterns predicted by the inverse dynamic model to those recorded during gait. EMG can be acquired by surface electrodes or by intramuscular measurements. Both methods have advantages and disadvantages. In this study surface EMG is used to minimise the influence of the measurements on normal gait as EMG is not used as an input for the model, only as a validation tool.

During the motion analysis force measurements are performed in 3D by a force plate and in one dimension by the plantar pressure plate. The force plate measures a global force vector of the ground reaction force, averaged in space. A pressure plate measures the individual vertical component of the ground reaction force over the full plantar surface. Ideally these measurements should be combined, yielding a high resolution measurement of three dimensional force vectors. Attempts have been made to develop this type of device, however no suitable and validated system is currently commercially available.

Weight bearing CT scans are performed with approximately 50% body weight. This is lower than would be encountered during normal gait, however the restrictions of the scanner mean that the participant needs to be in a sitting position so that the full foot and ankle can be imaged [36]. Commean et al [8] have demonstrated the reliability of weight-bearing CT in a sitting position.

Despite these limitations, the protocol will produce unique datasets consisting of detailed anatomic and dynamic measurements. These will be used to develop scalable biomechanical foot models to improve understanding of foot function and to attempt to predict the effect of foot and ankle orthotic design so that the design of these devices can be optimised prior to manufacture, potentially removing some of the variability in form and function that is seen with currently prescribed devices. Driving the models using dynamic plantar pressure rather than kinematic measurements could make this approach particularly useful and accessible for clinicians, avoiding the cost and time associated with full motion capture analysis. If validation of the models is successful, the next step will be to run a clinical trial to test if the use of the model for the development of foot and ankle orthoses leads to improved efficacy.

## Competing interests

Tørholm is joint founder of AnyBody Technology, a commercial developer of biomechanical modelling software. It is planned that the foot model will be made available through the company's repository of biomechanical models. SC is employed by AnyBody Technology.

## Authors' contributions

MO and Telfer will be responsible for data collection and prepared the initial draft of this manuscript. The original concept for this study was conceived by JW, Tørholm and LWvR as part of the A-FOOTPRINT project. SC has been responsible for the coupling of the modelling with the inverse dynamic model. RM has contributed to the imaging protocol. KM has contributed to the gait analysis protocol. All authors contributed to the development of the protocol and approved the final draft of this manuscript.

## Pre-publication history

The pre-publication history for this paper can be accessed here:

http://www.biomedcentral.com/1471-2474/12/256/prepub
